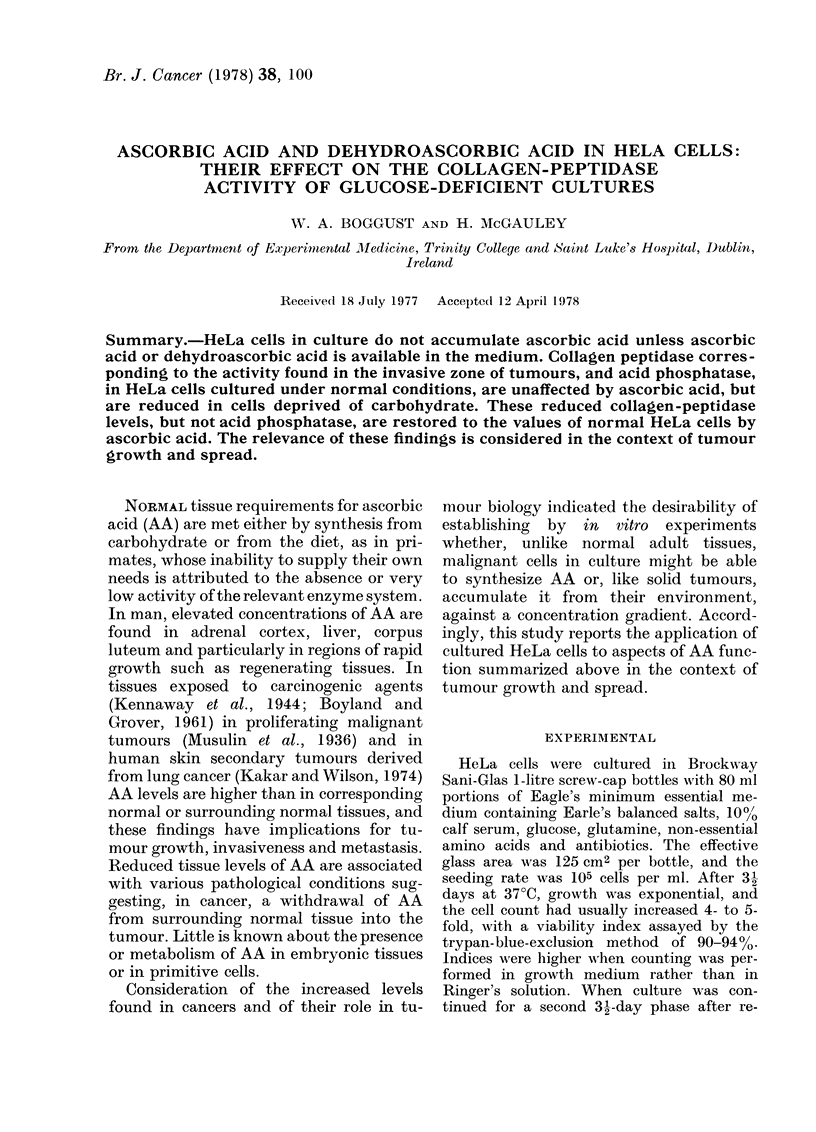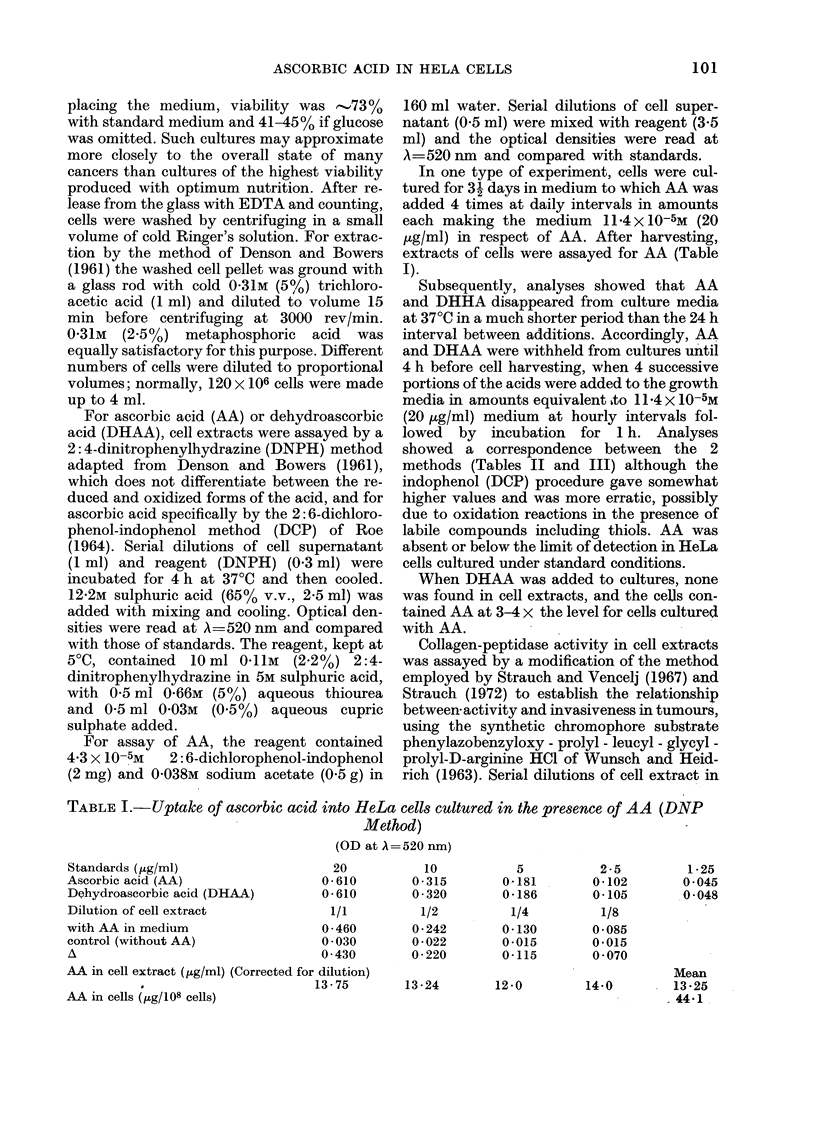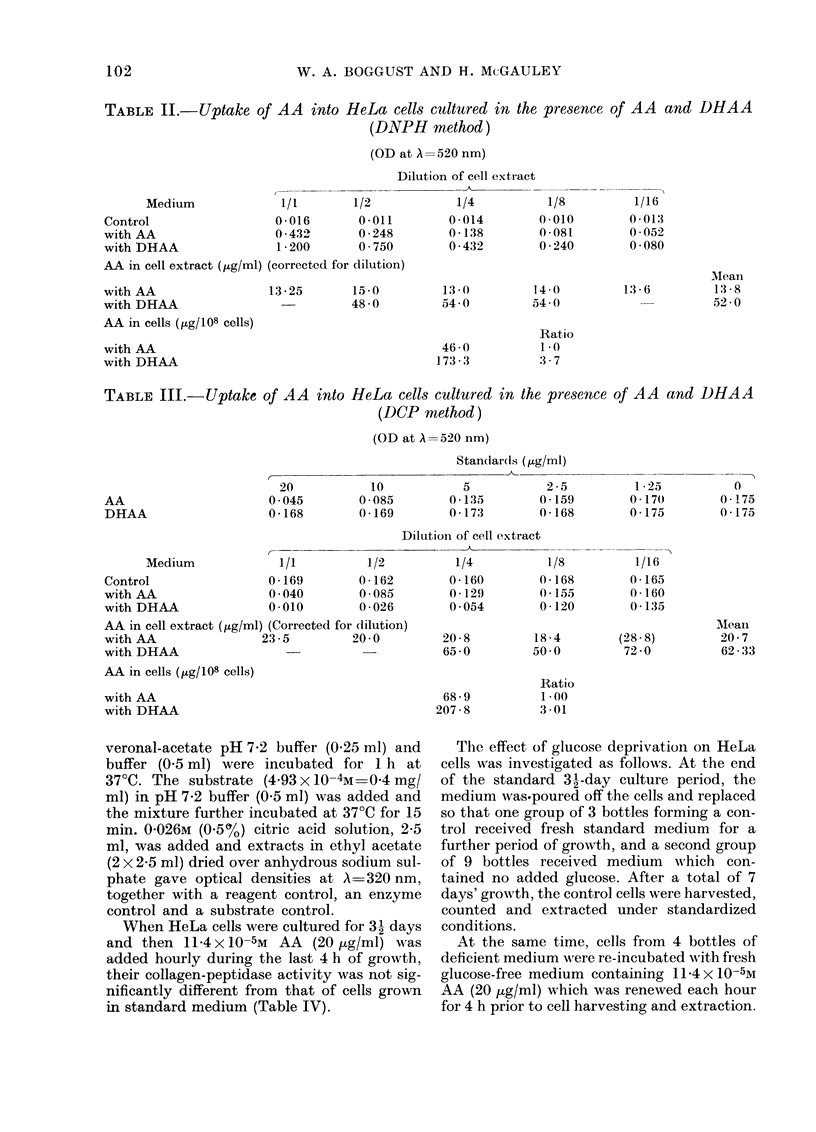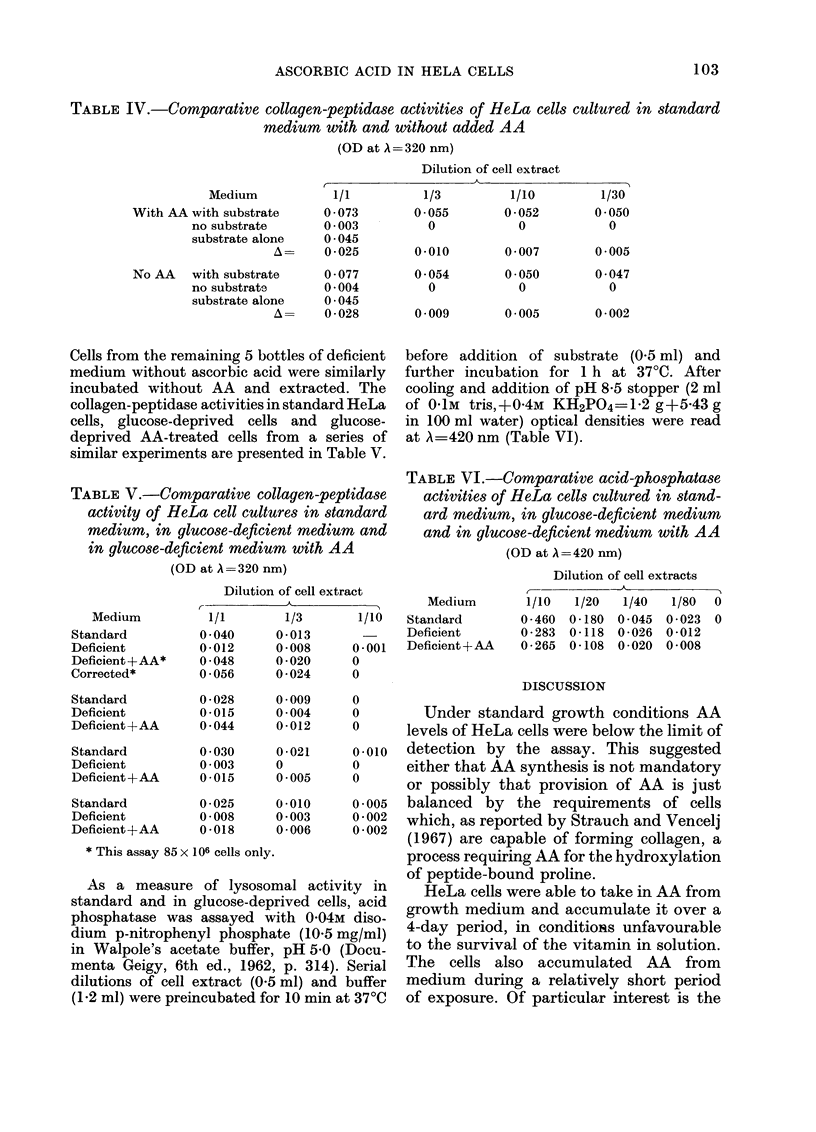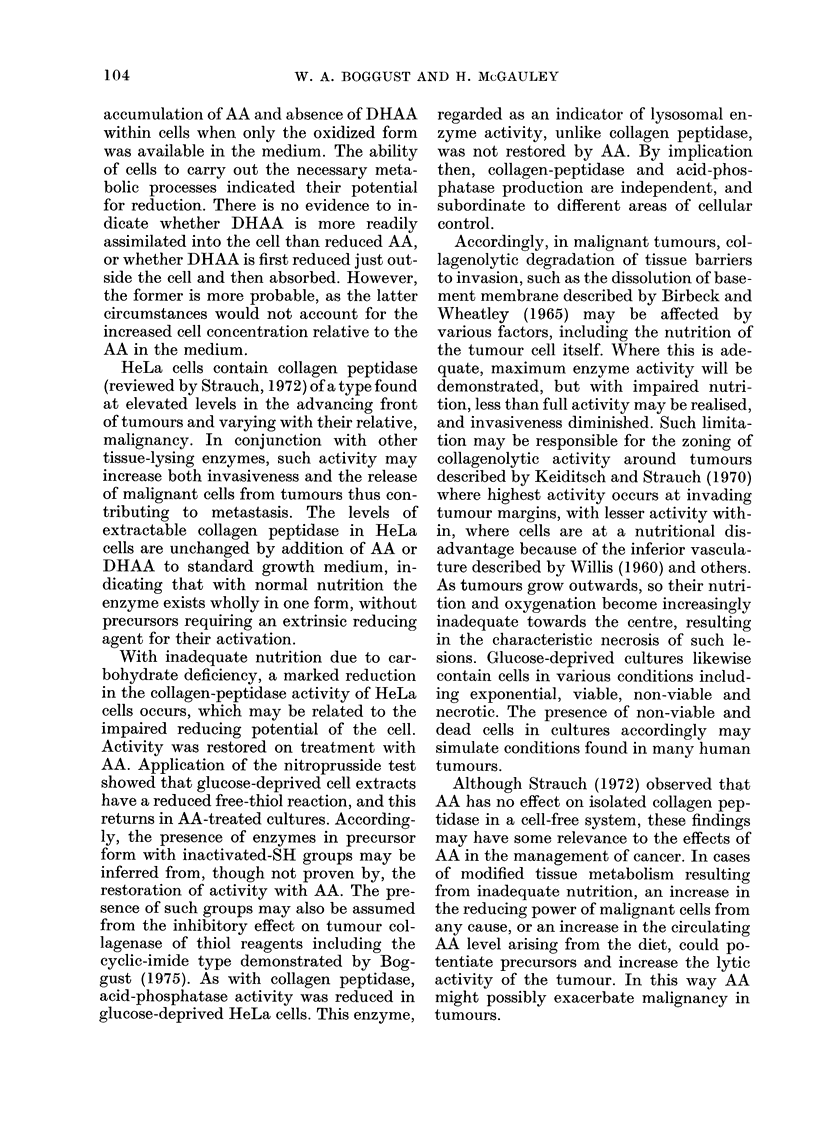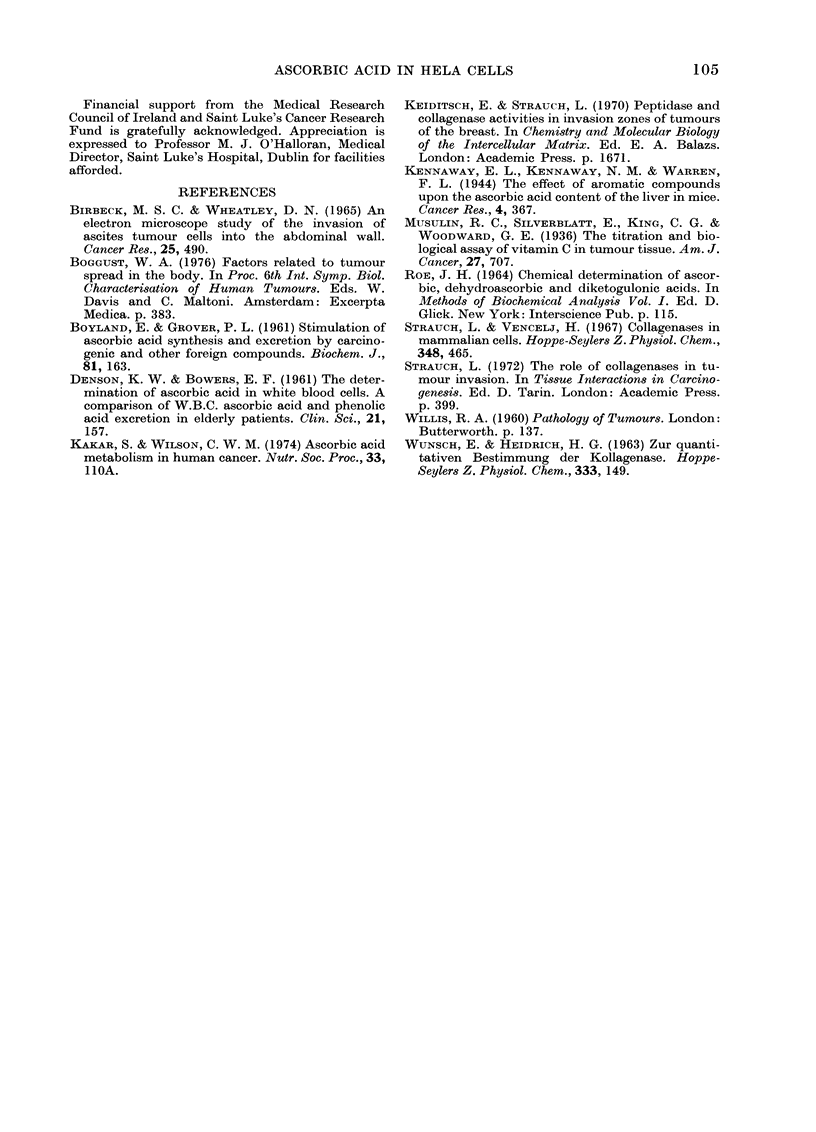# Ascorbic acid and dehydroascorbic acid in HeLa cells: their effect on the collagen-peptidase activity of glucose-deficient cultures.

**DOI:** 10.1038/bjc.1978.168

**Published:** 1978-07

**Authors:** W. A. Boggust, H. McGauley

## Abstract

HeLa cells in culture do not accumulate ascorbic acid unless ascorbic acid or dehydroascorbic acid is available in the medium. Collagen peptidase corresponding to the activity found in the invasive zone of tumours, and acid phosphatase, in HeLa cells cultured under normal conditions, are unaffected by ascorbic acid, but are reduced in cells deprived of carbohydrate. These reduced collagen-peptidase levels, but not acid phosphatase, are restored to the values of normal HeLa cells by ascorbic acid. The relevance of these findings is considered in the context of tumour growth and spread.


*Br. J. Cancer* (1978) **38**, 100

### ASCORBIC ACID AND DEHYDROASCORBIC ACID IN HELA CELLS: THEIR EFFECT ON THE COLLAGEN-PEPTIDASE ACTIVITY OF GLUCOSE-DEFICIENT CULTURES

W. A. BOGGUST AND H. McGAULEY

*From the Department of Experimental Medicine, Trinity College and Saint Luke's Hospital, Dublin, Ireland*

Received 18 July 1977 Accepted 12 April 1978

**Summary.—HeLa cells in culture do not accumulate ascorbic acid unless ascorbic acid or dehydroascorbic acid is available in the medium. Collagen peptidase corresponding to the activity found in the invasive zone of tumours, and acid phosphatase, in HeLa cells cultured under normal conditions, are unaffected by ascorbic acid, but are reduced in cells deprived of carbohydrate. These reduced collagen-peptidase levels, but not acid phosphatase, are restored to the values of normal HeLa cells by ascorbic acid. The relevance of these findings is considered in the context of tumour growth and spread.**

NORMAL tissue requirements for ascorbic acid (AA) are met either by synthesis from carbohydrate or from the diet, as in primates, whose inability to supply their own needs is attributed to the absence or very low activity of the relevant enzyme system. In man, elevated concentrations of AA are found in adrenal cortex, liver, corpus luteum and particularly in regions of rapid growth such as regenerating tissues. In tissues exposed to carcinogenic agents (Kennaway *et al.*, 1944; Boyland and Grover, 1961) in proliferating malignant tumours (Musulin *et al.*, 1936) and in human skin secondary tumours derived from lung cancer (Kakar and Wilson, 1974) AA levels are higher than in corresponding normal or surrounding normal tissues, and these findings have implications for tumour growth, invasiveness and metastasis. Reduced tissue levels of AA are associated with various pathological conditions suggesting, in cancer, a withdrawal of AA from surrounding normal tissue into the tumour. Little is known about the presence or metabolism of AA in embryonic tissues or in primitive cells.

Consideration of the increased levels found in cancers and of their role in tumour biology indicated the desirability of establishing by *in vitro* experiments whether, unlike normal adult tissues, malignant cells in culture might be able to synthesize AA or, like solid tumours, accumulate it from their environment, against a concentration gradient. Accordingly, this study reports the application of cultured HeLa cells to aspects of AA function summarized above in the context of tumour growth and spread.

### EXPERIMENTAL

HeLa cells were cultured in Brockway Sani-Glas 1-litre screw-cap bottles with 80 ml portions of Eagle's minimum essential medium containing Earle's balanced salts, 10% calf serum, glucose, glutamine, non-essential amino acids and antibiotics. The effective glass area was 125 $cm^2$ per bottle, and the seeding rate was $10^5$ cells per ml. After $3\frac{1}{2}$ days at 37°C, growth was exponential, and the cell count had usually increased 4- to 5-fold, with a viability index assayed by the trypan-blue-exclusion method of 90–94%. Indices were higher when counting was performed in growth medium rather than in Ringer's solution. When culture was continued for a second $3\frac{1}{2}$-day phase after replacing the medium, viability was ~73% with standard medium and 41–45% if glucose was omitted. Such cultures may approximate more closely to the overall state of many cancers than cultures of the highest viability produced with optimum nutrition. After release from the glass with EDTA and counting, cells were washed by centrifuging in a small volume of cold Ringer's solution. For extraction by the method of Denson and Bowers (1961) the washed cell pellet was ground with a glass rod with cold 0·31M (5%) trichloroacetic acid (1 ml) and diluted to volume 15 min before centrifuging at 3000 rev/min. 0·31M (2·5%) metaphosphoric acid was equally satisfactory for this purpose. Different numbers of cells were diluted to proportional volumes; normally, $120 \times 10^6$ cells were made up to 4 ml.

For ascorbic acid (AA) or dehydroascorbic acid (DHAA), cell extracts were assayed by a 2:4-dinitrophenylhydrazine (DNPH) method adapted from Denson and Bowers (1961), which does not differentiate between the reduced and oxidized forms of the acid, and for ascorbic acid specifically by the 2:6-dichlorophenol-indophenol method (DCP) of Roe (1964). Serial dilutions of cell supernatant (1 ml) and reagent (DNPH) (0·3 ml) were incubated for 4 h at 37°C and then cooled. 12·2M sulphuric acid (65% v.v., 2·5 ml) was added with mixing and cooling. Optical densities were read at $\lambda = 520$ nm and compared with those of standards. The reagent, kept at 5°C, contained 10 ml 0·11M (2·2%) 2:4-dinitrophenylhydrazine in 5M sulphuric acid, with 0·5 ml 0·66M (5%) aqueous thiourea and 0·5 ml 0·03M (0·5%) aqueous cupric sulphate added.

For assay of AA, the reagent contained $4 \cdot 3 \times 10^{-5}$M 2:6-dichlorophenol-indophenol (2 mg) and 0·038M sodium acetate (0·5 g) in 160 ml water. Serial dilutions of cell supernatant (0·5 ml) were mixed with reagent (3·5 ml) and the optical densities were read at $\lambda = 520$ nm and compared with standards.

In one type of experiment, cells were cultured for 3½ days in medium to which AA was added 4 times at daily intervals in amounts each making the medium $11 \cdot 4 \times 10^{-5}$M (20 μg/ml) in respect of AA. After harvesting, extracts of cells were assayed for AA (Table I).

Subsequently, analyses showed that AA and DHHA disappeared from culture media at 37°C in a much shorter period than the 24 h interval between additions. Accordingly, AA and DHAA were withheld from cultures until 4 h before cell harvesting, when 4 successive portions of the acids were added to the growth media in amounts equivalent to $11 \cdot 4 \times 10^{-5}$M (20 μg/ml) medium at hourly intervals followed by incubation for 1 h. Analyses showed a correspondence between the 2 methods (Tables II and III) although the indophenol (DCP) procedure gave somewhat higher values and was more erratic, possibly due to oxidation reactions in the presence of labile compounds including thiols. AA was absent or below the limit of detection in HeLa cells cultured under standard conditions.

When DHAA was added to cultures, none was found in cell extracts, and the cells contained AA at 3–4× the level for cells cultured with AA.

Collagen-peptidase activity in cell extracts was assayed by a modification of the method employed by Strauch and Vencelj (1967) and Strauch (1972) to establish the relationship between activity and invasiveness in tumours, using the synthetic chromophore substrate phenylazobenzyloxy - prolyl - leucyl - glycyl - prolyl-D-arginine HCl of Wunsch and Heidrich (1963). Serial dilutions of cell extract in

TABLE I.—*Uptake of ascorbic acid into HeLa cells cultured in the presence of AA (DNP Method)*

(OD at $\lambda = 520$ nm)

| | | | | | |
|---|---|---|---|---|---|
| Standards (μg/ml) | 20 | 10 | 5 | 2·5 | 1·25 |
| Ascorbic acid (AA) | 0·610 | 0·315 | 0·181 | 0·102 | 0·045 |
| Dehydroascorbic acid (DHAA) | 0·610 | 0·320 | 0·186 | 0·105 | 0·048 |
| Dilution of cell extract | 1/1 | 1/2 | 1/4 | 1/8 | |
| with AA in medium | 0·460 | 0·242 | 0·130 | 0·085 | |
| control (without AA) | 0·030 | 0·022 | 0·015 | 0·015 | |
| Δ | 0·430 | 0·220 | 0·115 | 0·070 | |
| AA in cell extract (μg/ml) (Corrected for dilution) | | | | | Mean |
| | 13·75 | 13·24 | 12·0 | 14·0 | 13·25 |
| AA in cells (μg/$10^8$ cells) | | | | | 44·1 |

TABLE II.—*Uptake of AA into HeLa cells cultured in the presence of AA and DHAA (DNPH method)*

(OD at $\lambda = 520$ nm)

| | Dilution of cell extract | | | | | |
|---|---|---|---|---|---|---|
| Medium | 1/1 | 1/2 | 1/4 | 1/8 | 1/16 | |
| Control | 0·016 | 0·011 | 0·014 | 0·010 | 0·013 | |
| with AA | 0·432 | 0·248 | 0·138 | 0·081 | 0·052 | |
| with DHAA | 1·200 | 0·750 | 0·432 | 0·240 | 0·080 | |
| AA in cell extract ($\mu$g/ml) (corrected for dilution) | | | | | | Mean |
| with AA | 13·25 | 15·0 | 13·0 | 14·0 | 13·6 | 13·8 |
| with DHAA | — | 48·0 | 54·0 | 54·0 | — | 52·0 |
| AA in cells ($\mu$g/$10^8$ cells) | | | | Ratio | | |
| with AA | | | 46·0 | 1·0 | | |
| with DHAA | | | 173·3 | 3·7 | | |

TABLE III.—*Uptake of AA into HeLa cells cultured in the presence of AA and DHAA (DCP method)*

(OD at $\lambda = 520$ nm)

| | Standards ($\mu$g/ml) | | | | | |
|---|---|---|---|---|---|---|
| | 20 | 10 | 5 | 2·5 | 1·25 | 0 |
| AA | 0·045 | 0·085 | 0·135 | 0·159 | 0·170 | 0·175 |
| DHAA | 0·168 | 0·169 | 0·173 | 0·168 | 0·175 | 0·175 |

| | Dilution of cell extract | | | | | |
|---|---|---|---|---|---|---|
| Medium | 1/1 | 1/2 | 1/4 | 1/8 | 1/16 | |
| Control | 0·169 | 0·162 | 0·160 | 0·168 | 0·165 | |
| with AA | 0·040 | 0·085 | 0·129 | 0·155 | 0·160 | |
| with DHAA | 0·010 | 0·026 | 0·054 | 0·120 | 0·135 | |
| AA in cell extract ($\mu$g/ml) (Corrected for dilution) | | | | | | Mean |
| with AA | 23·5 | 20·0 | 20·8 | 18·4 | (28·8) | 20·7 |
| with DHAA | — | — | 65·0 | 50·0 | 72·0 | 62·33 |
| AA in cells ($\mu$g/$10^8$ cells) | | | | Ratio | | |
| with AA | | | 68·9 | 1·00 | | |
| with DHAA | | | 207·8 | 3·01 | | |

veronal-acetate pH 7·2 buffer (0·25 ml) and buffer (0·5 ml) were incubated for 1 h at 37°C. The substrate ($4{\cdot}93 \times 10^{-4}$M$=0{\cdot}4$ mg/ml) in pH 7·2 buffer (0·5 ml) was added and the mixture further incubated at 37°C for 15 min. 0·026M (0·5%) citric acid solution, 2·5 ml, was added and extracts in ethyl acetate ($2 \times 2{\cdot}5$ ml) dried over anhydrous sodium sulphate gave optical densities at $\lambda = 320$ nm, together with a reagent control, an enzyme control and a substrate control.

When HeLa cells were cultured for 3½ days and then $11{\cdot}4 \times 10^{-5}$M AA (20 $\mu$g/ml) was added hourly during the last 4 h of growth, their collagen-peptidase activity was not significantly different from that of cells grown in standard medium (Table IV).

The effect of glucose deprivation on HeLa cells was investigated as follows. At the end of the standard 3½-day culture period, the medium was poured off the cells and replaced so that one group of 3 bottles forming a control received fresh standard medium for a further period of growth, and a second group of 9 bottles received medium which contained no added glucose. After a total of 7 days' growth, the control cells were harvested, counted and extracted under standardized conditions.

At the same time, cells from 4 bottles of deficient medium were re-incubated with fresh glucose-free medium containing $11{\cdot}4 \times 10^{-5}$M AA (20 $\mu$g/ml) which was renewed each hour for 4 h prior to cell harvesting and extraction.

TABLE IV.—*Comparative collagen-peptidase activities of HeLa cells cultured in standard medium with and without added AA*

(OD at $\lambda=320$ nm)

| Medium | | Dilution of cell extract 1/1 | 1/3 | 1/10 | 1/30 |
|---|---|---|---|---|---|
| With AA | with substrate | 0·073 | 0·055 | 0·052 | 0·050 |
| | no substrate | 0·003 | 0 | 0 | 0 |
| | substrate alone | 0·045 | | | |
| | Δ= | 0·025 | 0·010 | 0·007 | 0·005 |
| No AA | with substrate | 0·077 | 0·054 | 0·050 | 0·047 |
| | no substrate | 0·004 | 0 | 0 | 0 |
| | substrate alone | 0·045 | | | |
| | Δ= | 0·028 | 0·009 | 0·005 | 0·002 |

Cells from the remaining 5 bottles of deficient medium without ascorbic acid were similarly incubated without AA and extracted. The collagen-peptidase activities in standard HeLa cells, glucose-deprived cells and glucose-deprived AA-treated cells from a series of similar experiments are presented in Table V.

TABLE V.—*Comparative collagen-peptidase activity of HeLa cell cultures in standard medium, in glucose-deficient medium and in glucose-deficient medium with AA*

(OD at $\lambda=320$ nm)

| Medium | Dilution of cell extract 1/1 | 1/3 | 1/10 |
|---|---|---|---|
| Standard | 0·040 | 0·013 | — |
| Deficient | 0·012 | 0·008 | 0·001 |
| Deficient+AA* | 0·048 | 0·020 | 0 |
| Corrected* | 0·056 | 0·024 | 0 |
| Standard | 0·028 | 0·009 | 0 |
| Deficient | 0·015 | 0·004 | 0 |
| Deficient+AA | 0·044 | 0·012 | 0 |
| Standard | 0·030 | 0·021 | 0·010 |
| Deficient | 0·003 | 0 | 0 |
| Deficient+AA | 0·015 | 0·005 | 0 |
| Standard | 0·025 | 0·010 | 0·005 |
| Deficient | 0·008 | 0·003 | 0·002 |
| Deficient+AA | 0·018 | 0·006 | 0·002 |

* This assay $85\times10^6$ cells only.

As a measure of lysosomal activity in standard and in glucose-deprived cells, acid phosphatase was assayed with 0·04M disodium p-nitrophenyl phosphate (10·5 mg/ml) in Walpole's acetate buffer, pH 5·0 (Documenta Geigy, 6th ed., 1962, p. 314). Serial dilutions of cell extract (0·5 ml) and buffer (1·2 ml) were preincubated for 10 min at 37°C before addition of substrate (0·5 ml) and further incubation for 1 h at 37°C. After cooling and addition of pH 8·5 stopper (2 ml of 0·1M tris,+0·4M $KH_2PO_4$=1·2 g+5·43 g in 100 ml water) optical densities were read at $\lambda=420$ nm (Table VI).

TABLE VI.—*Comparative acid-phosphatase activities of HeLa cells cultured in standard medium, in glucose-deficient medium and in glucose-deficient medium with AA*

(OD at $\lambda=420$ nm)

| Medium | Dilution of cell extracts 1/10 | 1/20 | 1/40 | 1/80 | 0 |
|---|---|---|---|---|---|
| Standard | 0·460 | 0·180 | 0·045 | 0·023 | 0 |
| Deficient | 0·283 | 0·118 | 0·026 | 0·012 | |
| Deficient+AA | 0·265 | 0·108 | 0·020 | 0·008 | |

### DISCUSSION

Under standard growth conditions AA levels of HeLa cells were below the limit of detection by the assay. This suggested either that AA synthesis is not mandatory or possibly that provision of AA is just balanced by the requirements of cells which, as reported by Strauch and Vencelj (1967) are capable of forming collagen, a process requiring AA for the hydroxylation of peptide-bound proline.

HeLa cells were able to take in AA from growth medium and accumulate it over a 4-day period, in conditions unfavourable to the survival of the vitamin in solution. The cells also accumulated AA from medium during a relatively short period of exposure. Of particular interest is the accumulation of AA and absence of DHAA within cells when only the oxidized form was available in the medium. The ability of cells to carry out the necessary metabolic processes indicated their potential for reduction. There is no evidence to indicate whether DHAA is more readily assimilated into the cell than reduced AA, or whether DHAA is first reduced just outside the cell and then absorbed. However, the former is more probable, as the latter circumstances would not account for the increased cell concentration relative to the AA in the medium.

HeLa cells contain collagen peptidase (reviewed by Strauch, 1972) of a type found at elevated levels in the advancing front of tumours and varying with their relative, malignancy. In conjunction with other tissue-lysing enzymes, such activity may increase both invasiveness and the release of malignant cells from tumours thus contributing to metastasis. The levels of extractable collagen peptidase in HeLa cells are unchanged by addition of AA or DHAA to standard growth medium, indicating that with normal nutrition the enzyme exists wholly in one form, without precursors requiring an extrinsic reducing agent for their activation.

With inadequate nutrition due to carbohydrate deficiency, a marked reduction in the collagen-peptidase activity of HeLa cells occurs, which may be related to the impaired reducing potential of the cell. Activity was restored on treatment with AA. Application of the nitroprusside test showed that glucose-deprived cell extracts have a reduced free-thiol reaction, and this returns in AA-treated cultures. Accordingly, the presence of enzymes in precursor form with inactivated-SH groups may be inferred from, though not proven by, the restoration of activity with AA. The presence of such groups may also be assumed from the inhibitory effect on tumour collagenase of thiol reagents including the cyclic-imide type demonstrated by Boggust (1975). As with collagen peptidase, acid-phosphatase activity was reduced in glucose-deprived HeLa cells. This enzyme, regarded as an indicator of lysosomal enzyme activity, unlike collagen peptidase, was not restored by AA. By implication then, collagen-peptidase and acid-phosphatase production are independent, and subordinate to different areas of cellular control.

Accordingly, in malignant tumours, collagenolytic degradation of tissue barriers to invasion, such as the dissolution of basement membrane described by Birbeck and Wheatley (1965) may be affected by various factors, including the nutrition of the tumour cell itself. Where this is adequate, maximum enzyme activity will be demonstrated, but with impaired nutrition, less than full activity may be realised, and invasiveness diminished. Such limitation may be responsible for the zoning of collagenolytic activity around tumours described by Keiditsch and Strauch (1970) where highest activity occurs at invading tumour margins, with lesser activity within, where cells are at a nutritional disadvantage because of the inferior vasculature described by Willis (1960) and others. As tumours grow outwards, so their nutrition and oxygenation become increasingly inadequate towards the centre, resulting in the characteristic necrosis of such lesions. Glucose-deprived cultures likewise contain cells in various conditions including exponential, viable, non-viable and necrotic. The presence of non-viable and dead cells in cultures accordingly may simulate conditions found in many human tumours.

Although Strauch (1972) observed that AA has no effect on isolated collagen peptidase in a cell-free system, these findings may have some relevance to the effects of AA in the management of cancer. In cases of modified tissue metabolism resulting from inadequate nutrition, an increase in the reducing power of malignant cells from any cause, or an increase in the circulating AA level arising from the diet, could potentiate precursors and increase the lytic activity of the tumour. In this way AA might possibly exacerbate malignancy in tumours.

Financial support from the Medical Research Council of Ireland and Saint Luke's Cancer Research Fund is gratefully acknowledged. Appreciation is expressed to Professor M. J. O'Halloran, Medical Director, Saint Luke's Hospital, Dublin for facilities afforded.

### REFERENCES

BIRBECK, M. S. C. & WHEATLEY, D. N. (1965) An electron microscope study of the invasion of ascites tumour cells into the abdominal wall. *Cancer Res.*, **25**, 490.

BOGGUST, W. A. (1976) Factors related to tumour spread in the body. In *Proc. 6th Int. Symp. Biol. Characterisation of Human Tumours.* Eds. W. Davis and C. Maltoni. Amsterdam: Excerpta Medica. p. 383.

BOYLAND, E. & GROVER, P. L. (1961) Stimulation of ascorbic acid synthesis and excretion by carcinogenic and other foreign compounds. *Biochem. J.*, **81**, 163.

DENSON, K. W. & BOWERS, E. F. (1961) The determination of ascorbic acid in white blood cells. A comparison of W.B.C. ascorbic acid and phenolic acid excretion in elderly patients. *Clin. Sci.*, **21**, 157.

KAKAR, S. & WILSON, C. W. M. (1974) Ascorbic acid metabolism in human cancer. *Nutr. Soc. Proc.*, **33**, 110A.

KEIDITSCH, E. & STRAUCH, L. (1970) Peptidase and collagenase activities in invasion zones of tumours of the breast. In *Chemistry and Molecular Biology of the Intercellular Matrix.* Ed. E. A. Balazs. London: Academic Press. p. 1671.

KENNAWAY, E. L., KENNAWAY, N. M. & WARREN, F. L. (1944) The effect of aromatic compounds upon the ascorbic acid content of the liver in mice. *Cancer Res.*, **4**, 367.

MUSULIN, R. C., SILVERBLATT, E., KING, C. G. & WOODWARD, G. E. (1936) The titration and biological assay of vitamin C in tumour tissue. *Am. J. Cancer*, **27**, 707.

ROE, J. H. (1964) Chemical determination of ascorbic, dehydroascorbic and diketogulonic acids. In *Methods of Biochemical Analysis Vol. I.* Ed. D. Glick. New York: Interscience Pub. p. 115.

STRAUCH, L. & VENCELJ, H. (1967) Collagenases in mammalian cells. *Hoppe-Seylers Z. Physiol. Chem.*, **348**, 465.

STRAUCH, L. (1972) The role of collagenases in tumour invasion. In *Tissue Interactions in Carcinogenesis.* Ed. D. Tarin. London: Academic Press. p. 399.

WILLIS, R. A. (1960) *Pathology of Tumours.* London: Butterworth. p. 137.

WUNSCH, E. & HEIDRICH, H. G. (1963) Zur quantitativen Bestimmung der Kollagenase. *Hoppe-Seylers Z. Physiol. Chem.*, **333**, 149.